# Emergence of KPC-producing *Klebsiella pneumoniae *in Italy

**DOI:** 10.1186/1756-0500-3-40

**Published:** 2010-02-23

**Authors:** Carla Fontana, Marco Favaro, Loredana Sarmati, Silvia Natoli, Anna Altieri, Maria C Bossa, Silvia Minelli, Francesca Leonardis, Cartesio Favalli

**Affiliations:** 1 Department of Experimental Medicine and Biochemical Sciences, "Tor Vergata" University of Rome - Via Montpellier 1, Rome, 00133, Italy; 2 Clinical Microbiology Laboratories, Polyclinic of Tor Vergata, Viale Oxford 81, Rome, 00133, Italy; 3 Department of Surgery, Intensive Care Unit, Polyclinic Tor Vergata, Viale Oxford 81, Rome, 00133, Italy; 4 Infectious Diseases, Department of Public Health, Polyclinic of Tor Vergata, Viale Oxford 81, Rome, 00133, Italy

## Abstract

**Background:**

The emergence of KPC-producing *K. pneumoniae *has now become a global concern. KPC beta-lactamases are plasmid-borne and, like extended spectrum beta lactamases (ESBLs), can accumulate and transfer resistance determinants to other classes of antibiotics. Therefore, infection control guidelines on early identification and control of the spread of organisms carrying these resistant determinants are needed.

**Findings:**

*Klebsiella pneumoniae *carbapenemase (KPC) was detected in two isolates of carbapenem-resistant *K. pneumoniae *obtained from patients at an Italian teaching hospital. The first strain was isolated from a culture drawn from a central venous device (CVC) in a patient with Crohn's disease who was admitted to a gastroenterology ward. The second was isolated from a urine sample collected from an indwelling urinary catheter in an intensive care unit (ICU) patient with a subdural haematoma. The patients had not travelled abroad. Both isolates were resistant to all β-lactams and were susceptible to imipenem and meropenem but resistant to ertapenem. Isolates also showed resistance to other classes of non-β-lactam antibiotics, such as quinolones, aminoglycosides (with the exception for amikacin), trimethoprim-sulfamethoxazole (TMP-SMX) and nitrofurantoin. They were determined to contain the plasmid encoding the carbapenemase gene *bla-KPC *and were also positive in the Hodge test.

**Conclusions:**

This is the second report of KPC-producing isolates in Italy, but the first concerning KPC type 2 gene, and it may have important implications for controlling the transmission of microorganisms resistant to antibiotics.

## Findings

*Klebsiella pneumoniae *carbapenemase (KPC) is a molecular class A serine β-lactamase belonging to functional group 2f [1]. KPC was first reported in 2001 after its discovery in a *K. pneumoniae *clinical isolate collected in North Carolina during the ICARE (Intensive Care Antimicrobial Resistance Epidemiology) surveillance study [1-3]. The first detection of KPC-2 on a plasmid in *P. aeruginosa *was reported; this represents a disturbing development in the spread of these carbapenemases [2,4]. Although the KPC β-lactamases are predominantly found in *K. pneumoniae*, there have been reports of these enzymes in *Enterobacter *spp. and in *Salmonella *spp and in other genera of the Enterobacteriaceae family [5-10].

KPC-positive strains are typically resistant to the penicillins, extended-spectrum cephalosporins, and aztreonam, but the MICs of these drugs and of the carbapenems are reduced in the presence of clavulanic acid due to enzyme inhibition [2]. Isolates that acquire this enzyme are usually resistant to several other classes of antimicrobial agents used as treatment options. Laboratory identification of KPC-producing clinical isolates will be critical for limiting the spread of this resistance mechanism. The failure of automated susceptibility testing systems to detect KPC-mediated resistance was previously noted, particularly if ertapenem, which was determined to be more susceptible to the idrolytic activity of KPC carbanemase, was not tested [11]. After the rapid expansion of the KPC class of carbapenemases along the east coast of the United States, reports from across the world began to appear. A report from France in 2005 documented KPC-2 in a *K. pneumoniae *strain from a patient who had been in New York for medical treatment [12]. KPC-producing organisms have continued to spread over time and have now been reported in 27 states in the United States and in many countries around the world, including China, Colombia, Brazil, Israel, Canada, France, the Republic of Ireland, Greece (where infection caused by KPC is mainly due to hyperepidemic clone) and more recently in Italy [8-19]. We report, in this paper, the first two cases of KPC2 producing *K. pneumoniae *in Italy. Our finding confirms that KPC producing isolates are a global concern and that every effort should be made from the laboratory to early identification of these phenotypes.

## Case Presentation

### Case 1

A 48-year-old man was admitted to Tor Vergata University hospital in February 2009). He had suffered from Crohn's disease since 1990, with prolonged illness exacerbation periods. He received numerous treatments and had recently decided to treat himself with steroids. A colonoscopy was performed to remove a colic polyp, but an intra-abdominal perforation complicated the exam. A stercoraceous peritonitis developed and the patient urgently underwent intestinal resection and splenectomy. Seven days later, an ileal necrosis of the colostomy tract appeared and he underwent a resection of ileum. A few days later, he developed a fever and leucocitosis, and a Computed Tomography (CT) scan revealed an abdominal abscess that was drained. Different bacterial isolates were obtained from the blood and abscess drainage cultures (*Enterococcus faecalis, Escherichia coli, Candida albicans, Klebsiella pneumoniae *and *Acineobacter baumannii*), *K. pneumoniae *was cultured from the tip of a CVC (which was confirmed as KPC producing in two days). Numerous antibiotic treatments were used. The initial drug regimen consisted of linezolid, amikacin, caspofungin, piperacillin-tazobactam, metronidazole and then, after the identification of a KPC-producing *K. pneumoniae *(due to the positivity of the Hodge's test), the antibiotic therapy was shifted to an association of tigecycline (100 mg for the first dose and then 50 mg every 12 h) and amikacin (1 g once a day) for 9 days. At the time of multi-drug resistant *A. baumannii *isolation, amikacin was suspended and tigecycline continued in combination with colistin (2.000.000 units every 8 h). The association of tigecycline and colistin was suspended after 16 days. The patient was discharged without fever, abdominal drainage was removed when nothing further was picked up.

### Case 2

A 79-year-old woman with an intra-cerebral haemorrhage was admitted to the neurosurgical department of Tor Vergata University hospital in March, 2009. She had lost consciousness following a sudden headache and subsequent head trauma. The patient was operated on immediately and was later admitted to the ICU. Her past history revealed chronic atrial fibrillation, so she was administered dicumarol. Moreover, in the past she had suffered from recurrent urinary tract infections treated by her family doctor with ciprofloxacin. On admission, the patient was intubated and a urinary catheter was inserted, a central intravenous catheter was placed in her subclavian vein, and an arterial line was also inserted. She started a course of therapy with piperacillin/tazobactam (4.5 g/i.v. q6h) for the treatment of aspiration pneumonia due to *P. aeruginosa*. On the ninth day, the patient's temperature increased to 39°C and she experienced chills. Her white blood count and C reactive protein also increased (11.300 mm^3 ^and 102 mg/L, respectively). Several blood cultures (from the CVC or from the peripheral vein), urine and bronchial secretions (BAS) were collected for cultures. An empirical treatment was started by adding teicoplanin (600 mg i.v. q24 h) and levofloxacin (500 mg i.v. q12h) to the previous antibiotic therapy. The blood cultures from the CVC resulted positive for *Proteus mirabilis *and *Enterococcus faecalis*. The culture of BAS evidenced the same *P. aeruginosa *previously isolated, while the culture of urines yielded 10^6 ^CFU/mL of *K. pneumoniae *KPC-producing (initially confirmed by the Hodge's test). The previous antibiotic treatment was altered to amikacin (1,5 g i.v. q24h), while teicoplanin was maintained at the same dose. In the meantime, CVC was removed. After eight days, there was a second episode of fever. The amikacin treatment was interrupted and a tigecycline regimen was initiated (100 mg for the first dose and then 50 mg q12h). Blood and urine samples were collected again and a single blood culture was positive for *P. aeruginosa*. After ten days of treatment with meropenem, the infectious event clinically resolved, but the patient's conditions slowly worsened. After 27 days of hospitalization, the patient died due to the progression of her brain damage.

## Methods

Isolates were tested for susceptibility using Vitek2 (bioMérieux, Durham, NC), using AST GN13 and AST GN074, as well as the AST N022 card. Susceptibility was also confirmed for meropenem, imipenem, ertapenem, colistin and amikacin using Etest (bioMérieux Italia; Firenze, Italy) according to the manufacturer's recommendations and incubated for 18 hours at 35°C in ambient air. MICs were interpreted according to the Clinical Laboratory Standards Institute (CLSI) guidelines [20]. MIC for colistin were interpreted according to the EUCAST breakpoints available at the web site http://www.srga.org/eucastwt/MICTAB/index.html. Isolates were also tested using the modified Hodge test [21]. To exclude the possibility of a metallo-beta-lactamases (MBL) production a synergy test using imipenem and EDTA discs was used, the test was performed according to CLSI [20,22]

Analysis of β-lactamase genes (including *bla*_KPC _*bla*_TEM _*bla*_OXA_, and *bla*_SHV_), CTX-M type, VIM type, IMP type as well as *bla*AmpC were preformed as previously reported [13,23-26].

Amplified products of the expected size were confirmed as KPC by sequencing. For typing of the *bla*_KPC _gene, overlapping PCR reactions were performed using the following primer pairs: F-5'CGGAACCATTCGCTAAACTC3' and R-3'GGCGGCGTTATCACTGTATT5' primers; F-5'CGCCGTGCAATACAGTGATA3' and R-3'CGTTGACGCCCAATCC5' primers [13].

Amplification products were purified using the Montage PCR Centrifugal Filter Device (Millipore Corporation, Billerica, MA), and sequencing was performed by Big Dye Terminators V1.1 (Applied Biosystems, Foster City, CA) and migrated with an automated sequencer (ABI Prism 310; Applied Biosystems). Sequences were aligned and compared using the National Center for Biotechnology Information database http://www.ncbi.nlm.nih.gov/.

## Results

Isolates from case 1 and case 2 were both multi-drug resistant, being resistant to all β-lactam, quinoloni and aminoglycosides (with the exception of amikacin), TMP-SMX, and nitrofurantoin, and were susceptible to tigecycline and colistin (see Table 1). The behaviour versus carbapenemic compounds was typical for KPC isolates: both isolates were susceptible to imipenem (MIC = 1 μg/L) and meropenem (MIC = 2 μg/L) and resistant to ertapenem (MIC = 8 μg/L). Screening with ertapenem indicated the possibility of a KPC, which was confirmed by the modified Hodge test. While the MBL test was negative. Analysis of β-lactamase genes by PCR and sequencing revealed the presence of *bla*KPC-2, and *bla*SHV-1, while genes encoding other enzymes *bla*TEM, *bla*OXA, CTX-M type, VIM type, IMP type as well as *bla*AmpC were not detected.

**Table 1:** Susceptibility of KPC-producing *Klebsiella pneumoniae isolates*

*MICs (μg/ml)*																
***Isolates***	**AMP**	**AMC**	**PIP**	**PTZ**	**CAZ**	**CTX**	**FEP**	**IPM**	**MEM**	**EPT**	**AMK**	**CIP**	**TGC**	**CS**	**TMP-SMX**	**NT**

Case 1 isolate	32	32	128	128	16	64	16	1	2	8	2	8	0.5	0.5	320	512

Case 2 isolate	32	32	128	128	16	64	16	1	2	8	2	8	0.5	0.5	320	512

Abbreviations: AMP, ampicillin, AMC, amoxicillin-clavulanate; PIP, piperacillin, PTZ, piperacillin-tazobactam; CAZ, ceftazidime; CTX, cefotaxime, FEP, cefepime; IPM, imipenem; MEM, meropenem; EPT, ertapenem; AMK, amikacin; CIP, ciprofloxacin; TGC, tigecycline; CS, colistin; TMP-SMX, trimethoprim-sulfamethoxazole; NT, nitrofurantoin.

Both patients improved clinically with tigecycline treatment, even if for the second patient a septic event due to *P. aeruginosa *(successive to *K. pneumoniae *infections) needed to be treated with meropenem.

## Conclusion

This is the second documented appearance of a class A carbapenemase-producing isolate of *K. pneumoniae *in Italy, harbouring KPC type 2 gene, that was not associated with travel abroad. KPC β-lactamases (KPC 1-7) confer decreased susceptibility or resistance to all β-lactams [14,27]. The isolate showed reduced susceptibility or resistance to four different antibiotics, limiting the therapeutic options to polymixin, amikacin, and tigecycline [13]. At present, these are the second published cases of KPC-producing *K. pneumoniae *infections in Italy, after that published by Giani et al [19]. The dissemination of KPC-producing organisms is no longer restricted to the United States and has emerged as a global concern, aided by the recently described mobile genetic element Tn *4401*, which carries the KPC genes [28]. Carbapenemase-producing pathogens cause infections that are difficult to treat and have high mortality rates due to their appearance in multidrug-resistant pathogens such as *K. pneumoniae*, *P. aeruginosa*, and *Acinetobacter *spp [2]. The first descriptions of these enzymes as species-specific chromosomal carbapenemases have more recently been followed by the appearance of carbapenemase genes that are easily transferred on mobile elements between species. While considered by some to be relatively rare, reports of their occurrence in outbreak settings have steadily increased [2,13,14]. Detecting their entry into the hospital environment is the first step that clinical microbiologists can take to address this problem. Care in detection is needed, because high carbapenem MICs are not always evident and the phenotype could be confused with that described by Cagnacci et al [11,22,29]. Therefore, it is essential that the initial complete screening of the isolate includes Hodge's test as well as MBL test. Evaluation of effective antibiotic options and rigorous infection control measures will help in the fight against carbapenemase-producing organisms [2]. Regardless of the geographic location, microbiology laboratories in Italy need to ensure that they have methods in place for the accurate detection of KPC-producing organisms and must constantly be aware of their potential to spread.

## List of abbreviations used

KPC: *Klebsiella pneumoniae *carbapenemase; CVC: Central venousdevice; ICARE: Intensive Care Antimicrobial Resistance Epidemiology; ICU: Intensive care unit; TMP-SMX: Trimethoprim-sulfamethoxazole; CT: Computed Tomography; BAS: Bronchial secretions; CLSI: Clinical Laboratory Standards Institute; MBL: Metallo-beta-lactamases.

## Consent

Given the impossibility of finding the first patient (lost to follow up) and the death of the second, the ethics committee of the hospital gave approval for anonymous publication of these clinical cases (n 17/08 English version 04/2009).

## Competing interests

All authors declare no financial or personal relationships with other people or organizations that could inappropriately have influenced (bias) their work.

## Authors' contributions

CF and LS, SN and MF contributed to the conception of the study, in data analysis and are also involved in drafting the manuscript. FL and CsF contributed to the review of the study and data analysis. AA, MCB and SM contributed in acquisition and interpretation of data. All authors approved the final version of the manuscript.
